# Chondroprotective and anti-inflammatory effects of ChondroT, a new complex herbal medication

**DOI:** 10.1186/s12906-016-1211-0

**Published:** 2016-07-13

**Authors:** Jung Up Park, Seon-Jong Kim, Chang-Su Na, Chan-hun Choi, Chang Seob Seo, Jong-Keun Son, Bok Yun Kang, Young Ran Kim

**Affiliations:** College of Pharmacy and Research Institute of Drug Development, Chonnam National University, Gwangju, 500-757 Republic of Korea; College of Korean Medicine, Dongshin University, Naju-si, 58245 Republic of Korea; Korea Institute of Oriental Medicine, Daejeon, 34054 Republic of Korea; College of Pharmacy, Yeungnam University, Gyeongsan-si, Republic of Korea

**Keywords:** Anti-arthritic drug, ChondroT, Chondrocyte, MMP-1, COX-2, iNOS, Inflammatory cytokines

## Abstract

**Background:**

Ganghwaljetongyeum (GHJTY) is a complex herbal decoction comprising 18 plants; it is used to treat arthritis. In order to develop a new anti-arthritic herbal medication, we selected 5 out of 18 GHJTY plants by using bioinformatics analysis. The new medication, called ChondroT, comprised water extracts of Osterici Radix*,* Lonicerae Folium, Angelicae Gigantis Radix, Clematidis Radix, and Phellodendri Cortex*.* This study was designed to investigate its chondroprotective and anti-inflammatory effects to develop an anti-arthritic herb medicine.

**Methods:**

ChondroT was validated using a convenient and accurate high-performance liquid chromatography–photodiode array (HPLC–PDA) detection method for simultaneous determination of its seven reference components. The concentrations of the seven marker constituents were in the range of 0.81–5.46 mg/g. The chondroprotective effects were evaluated based on SW1353 chondrocytes and matrix metalloproteinase 1 (MMP1) expression. In addition, the anti-inflammatory effects of ChondroT were studied by Western blotting of pro-inflammatory enzymes and by enzyme-linked immunosorbent assay (ELISA) of inflammatory mediators in lipopolysaccharides (LPS)-induced RAW264.7 cells.

**Results:**

ChondroT enhanced the growth of SW1353 chondrocytes and also significantly inhibited IL-1β-induced MMP-1 expression. However, ChondroT did not show any effects on the growth of HeLa and RAW264.7 cells. The expression of cyclooxygenase-2 (COX-2) and inducible nitric oxide synthase (iNOS) was induced by LPS in RAW264.7 cells, which was significantly decreased by pre-treatment with ChondroT. In addition, ChondroT reduced the activation of NF-kB and production of inflammatory mediators, such as IL-1β, IL-6, PGE_2_, and nitric oxide (NO) in LPS-induced RAW264.7 cells.

**Conclusions:**

These results show that ChondroT exerted a chondroprotective effect and demonstrated multi-target mechanisms related to inflammation and arthritis. In addition, the suppressive effect was greater than that exhibited by GHJTY, suggesting that ChondroT, a new complex herbal medication, has therapeutic potential for the treatment of arthritis.

## Background

Arthritis is the most common inflammatory disease and a major public concern in elderly individuals. The symptoms include joint pain, tenderness, and joint inflammation. Although rheumatoid arthritis and osteoarthritis differ fundamentally in several respects, they result in cartilage degradation, which in turn leads to cartilage bone damage [1]. Matrix metalloproteases (MMPs) play a critical role in the breakdown of cartilage [2, 3]. In particular, MMP-1 is known to decompose type II collagen, which is a major component of chondrocytes [4]. Cartilage degradation in arthritis is recognized to be induced by inflammatory cytokines, such as interleukin (IL)-6, IL-1β, and tumor necrosis factor-α (TNF-α) [5–7]. Recently, prostaglandin E_2_ (PGE_2_) or nitric oxide (NO) has been shown to play key roles in the induction of MMP expression in chondrocytes [8, 9]. In addition to chondrocytes, macrophages contribute to inflammation and matrix degradation in osteoarthritis tissues, and inflammatory mediators such as IL-1β, TNF-α, IL-6, PGE_2_, and NO represent potential targets for osteoarthritis disease modification [9].

Proinflammatory enzymes such as cyclooxygenase-2 (COX-2) and inducible nitric oxide synthase (iNOS) that cause pain and inflammation, provide a measure to assess the effect of drugs for the treatment of arthritis [10]. Nonsteroidal anti-inflammatory drugs (NSAIDs) and selective COX-2 inhibitors are pharmacological treatments used for arthritis. Some oriental medicines have been used to treat arthritis [11–13]. We reported that Ganghwaljetongyeum (GHJTY), a traditional Korean herbal medicine used to treat severe joint pain, limitation of motion, fever, and swelling, inhibited inflammatory processes associated with arthritis [14]. Because GHJTY is a complex herbal decoction composed of 18 plants, we selected 5 effective herbs, i.e., Osterici Radix*,* Lonicerae Folium, Angelicae Gigantis Radix, Clematidis Radix, and Phellodendri Cortex, using bioinformatics analysis to develop a new anti-arthritic herbal medication [15]. In the present study, the water extracts of these 5 herbs named ChondroT was evaluated as an anti-arthritic herb drug. To develop a multi-functional herbal medicine for arthritis, we tested the effects of ChondroT on various arthritis-related pathomechanisms. The effects of ChondroT were evaluated on SW1353 chondrocyte protection and IL-1β-induced MMP1 expression. In addition, the inhibitory effects of ChondroT were studied on the expression of inflammatory enzymes COX-2 and iNOS and on the production of inflammatory mediators such as IL-1β, TNF-α, IL-6, PGE_2_, and NO in RAW264.7 macrophage cells.

## Methods

### Plant materials

The five herbal medicines forming ChondroT –Osterici Radix*,* Lonicerae Folium, Angelicae Gigantis Radix, Clematidis Radix, and Phellodendri Cortex – listed in Table 1 were purchased from Omniherb (Yeongcheon, Korea). The origin of the five herbal medicines was confirmed taxonomically by Professor Jong-Kil Jeong, Dept. of Herbology, College of Oriental Medicine, Dongshin University, Republic of Korea. Voucher specimens (KYR2014-020) have been deposited at college of Pharmacy, Chonnam National University.

**Table 1:** Composition of ChondroT

Latin name	Scientific name	Family	w/w %	Source
Osterici Radix	*Ostericum koreanum* Maximowicz	Umbelliferae	28.6	Korea
Lonicerae Folium	*Lonicera japonica* Thunberg	Caprifoliaceae	19.0	China
Angelicae Gigantis Radix	*Angelica gigas* Nakai	Umbelliferae	19.0	Korea
Clematidis Radix	*Clematis mandshurica* Ruprecht	Ranunculaceae	19.0	China
Phellodendri Cortex	*Phellodendron amurense* Ruprecht	Rutaceae	14.3	China

### Preparation of ChondroT

We combined 5 herbs containing Osterici Radix*,* Lonicerae Folium, Angelicae Gigantis Radix, Clematidis Radix, and Phellodendri Cortex in a ratio listed in Table 1. ChondroT herb material composed of above 5 herbs was extracted once using 10-fold water solvent at 100 °C for 3 h and then filtered (180 mesh). The water extract solution of ChondroT was concentrated using a continuous vacuum evaporator (around 55 ~ 60 °C, 670 mmHg) followed by lyophilization using a vacuum drier (720 mmHg) for 8 h. The water extract from GHJTY herbs was prepared as previously described [14]. Stock solutions of ChondroT and GHJTY were prepared in a concentration of 50 mg/mL using phosphate buffered saline (PBS) and filter-steriled.

### Reagents and high-performance liquid chromatography (HPLC) analysis

**Fig. 1:**
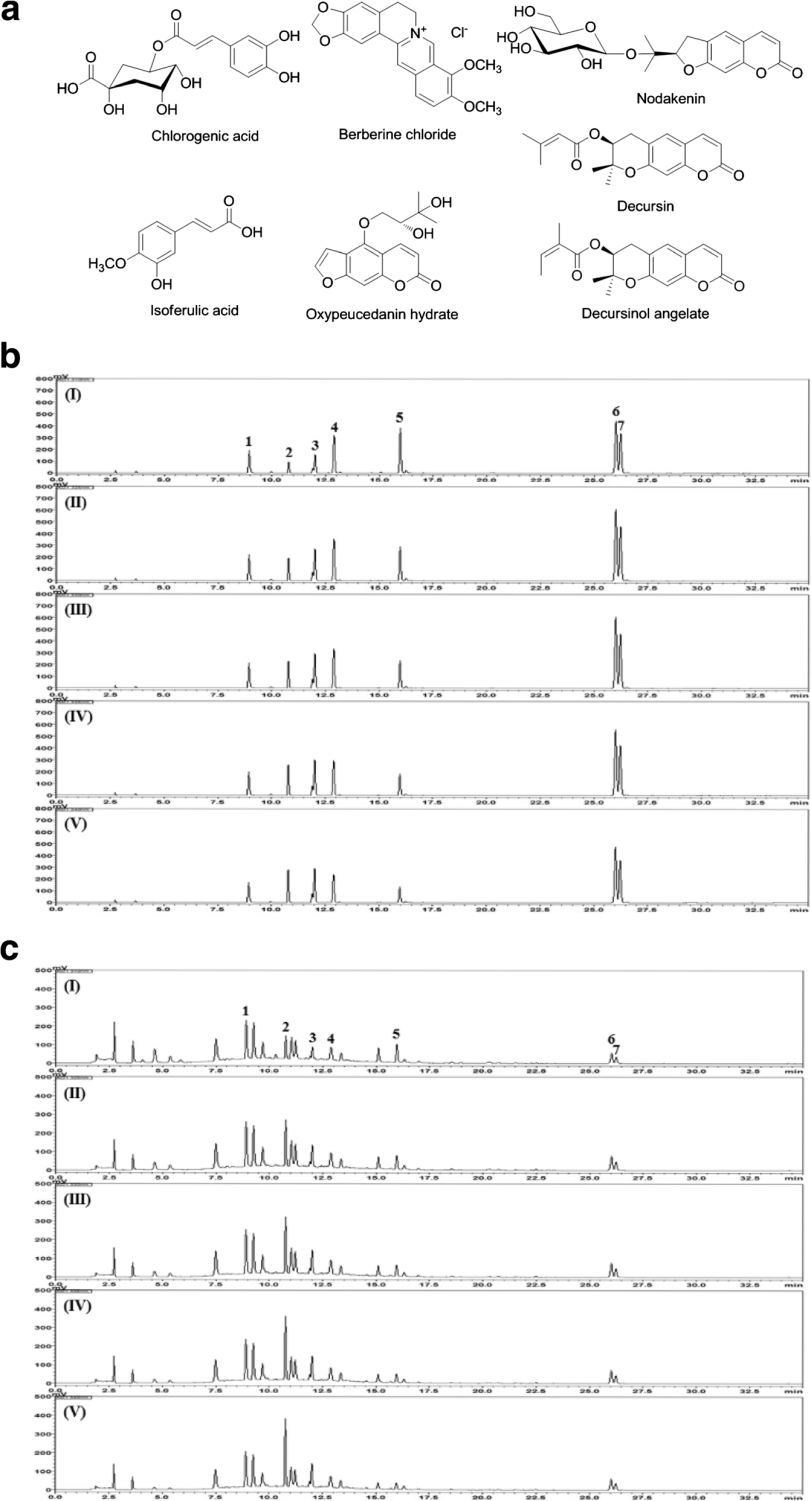
Chemical structure of the seven marker compounds (**a**) and HPLC chromatogram of a standard solution (**b**) and ChondroT (**c**) with detection at 310 nm (I), 325 nm (II), 330 nm (III), 335 nm, and 340 nm (V). Chlorogenic acid (*1*), berberine Cl (*2*), nodakenin (*3*), isoferulic acid (*4*), oxypeucedanin hydrate (*5*), decursin (*6*), and decursinol angelate (*7*)

Seven reference compounds used for quality control of ChondroT are shown in Table 2. The chemical structures of the seven marker compounds are shown in Fig. 1a. HPLC-grade solvents, methanol, acetonitrile, and water were obtained from J.T. Baker (Phillipsburg, NJ, USA). Analytical grade formic acid was purchased from Sigma-Aldrich (St. Louis, MO, USA). All reference compounds were dissolved in methanol at 1.0 mg/mL and stored at 4 °C. Working standard solutions were prepared by serial dilution of the stock solutions with methanol. For HPLC analysis, lyophilized ChondroT (200 mg) was dissolved in 20 mL of 70 % methanol and extracted for 60 min by sonication. All the stock solutions and ChondroT extract were passed through a 0.2-μm syringe filter (Woongki Science, Seoul, Korea) before HPLC analysis. The chromatographic analysis was conducted using a Shimadzu Prominence LC-20A series system (Shimadzu, Kyoto, Japan) consisting of a solvent delivery unit (LC-20AT), online degasser (DGU-20A_3_), column oven (CTO-20A), auto sample injector (SIL-20 AC), and photodiode array (PDA) detector (SPD-M20A). Data were acquired and processed using Labsolution software (version 5.54 SP3, Shimadzu, Kyoto, Japan). The column used was Waters SunFire C_18_ (5 μm, 4.6 × 250 nm, Milifird, MA, USA). The flow rate was kept constant at 1.0 mL/min, while the column temperature was maintained at 40 °C, and the injection volume was 10 μL. The gradient elution of two mobile phase systems with 0.1 % (v/v) formic acid in water (solvent A) and 0.1 % (v/v) formic acid in acetonitrile (solvent B) was as follows: 10–100 % B for 0–30 min, 100 % B for 30–40 min, and 100–10 % B for 40–50 min, with a re-equilibrium time of 10 min.

**Table 2:** Reference compounds for quality control of ChondroT

Compound	Purity (%)	Company	Detection wave length (nm)
Chlrogenic acid	≥ 99.0	Acros Organics (Pittsburgh, PA, USA)	325
Berberine Cl	≥ 98.0	Sigma-Aldrich Co. (St. Louis, MO, USA)	340
Nodakenin	≥ 98.0	NPC BioTechnology (Yeongi, Korea)	335
Isoferulic acid	≥ 98.0	ChemFaces (Wuhan, China)	325
Oxypeucedanin hydrate	≥ 98.0	ChemFaces (Wuhan, China)	310
Decursin	≥ 98.0	NPC BioTechnology (Yeongi, Korea)	330
Decursinol angelate	≥ 98.0	NPC BioTechnology (Yeongi, Korea)	330

### Cell cultures

SW1353 chondrosarcoma cells, RAW264.7 macrophage cells, and 293T human kidney epithelial cells were purchased from American Type Culture Collection (Manassas, VA, USA) and HeLa human cervix epithelial cells were supplied from Korea Cell Line Bank (Korea). The cells were cultured in Dulbecco Modified Eagle’s medium (DMEM) (Welgene, Korea) supplemented with 1 % penicillin-streptomycin and 10 % fetal bovine serum (FBS) (Gibco BRL, Rockville, MD, USA) under an atmosphere of 5 % CO_2_ in a humidified 37 °C incubator.

### MTS assay for cell viability test

SW1353, HeLa, or RAW264.7 cells were seeded in 96 well plates (SPL life sciences Co., Pocheon, Korea) at 0.5–1.0 × 10^4^/well. The cells were treated with ChondroT or GHJTY for 48 h. Cell proliferation was assayed using 3-(4, 5-dimethylthiazol-2-yl)-5-(3-carboxymethoxyphenyl) - 2-(4-sulfophenyl)-2H-tetrazolium (MTS), according to manufacturer’s instructions (Promega, Madison, WI, USA). Absorbance was read with an ELISA microplate reader (ELx808) (BioTek Instruments, Inc., Winooski, VT, USA) at 490 nm.

### Western blot analysis of MMP1

Human SW1353 chondrosarcoma cells were cultured in a 6-well plate (SPL life sciences Co., Pocheon, Korea) at 5 × 10^5^/well for 24 h. The cells were pretreated with ChondroT or GHJTY (0.3 mg/mL) for 2 h and then IL-1β or PMA (10 ng/mL) (Sigma Co., St. Louis, MO, USA) was added to the cells for 24 h. Equal amounts of cell supernatants were concentrated by acetone followed by boiling in sample buffer (Bio-solution, Suwon, Korea) for 10 min. The samples were subjected to 12 % SDS-PAGE and electro-transferred onto a nitrocellulose membrane (Millipore, Bedford, MA, USA). The membrane was blocked with 5 % skim milk and probed with an MMP1 antibody (Santa Cruz Biotechnology Inc., Santa Cruz, CA, USA) and rabbit IgG-HRP second antibody (Dako, Japan). The blots were washed three times using Tris-buffered saline with 0.1 % Tween 20 (TBST) and visualized using enhanced electrochemiluminescent (ECL) Western blotting detection kit (Advansta Corp., Menlo Park, CA, USA). The relative amount of MMP1 protein was analyzed by azure c-300 (Azure Biosystems, CA, USA).

### Western blot analysis of COX-2 and iNOS

RAW264.7 cells were cultured in a 6-well plate at 1 × 10^6^/well for 4 h. The cells were pretreated with ChondroT, GHJTY, or celecoxib (20 μM) (Sigma Co., MO, USA) for 2 h and then LPS (500 ng/mL) (Sigma Co., MO, USA) was added to the cells for 24 h. Equal amounts of cell lysates (25 μg) were subjected to 10 % SDS-PAGE and electro-transferred onto polyvinylidene fluoride membranes (PVDF) (Millipore, Bedford, MA, USA). Western bot analysis was conducted using the above mentioned methods with polyclonal antibodies specific to COX-2 (Cell signaling Tech., Danvers, MA, USA), iNOS (Santa Cruz Biotechnology Inc., Santa Cruz, CA, USA), or GAPDH (Santa Cruz Biotechnology Inc., Santa Cruz, CA, USA).

### Enzyme-linked immunosorbent assay (ELISA) of proinflammatory cytokines

RAW264.7 cells were cultured at 1 × 10^5^/well in 48-well plates (SPL life sciences Co., Pocheon, Korea) for 24 h. The cells were washed with fresh medium and treated with ChondroT or GHJTY (1, 0.3, or 0.1 mg/mL) for 2 h, followed by treatment with 500 ng/mL LPS (Sigma Co., MO, USA) for 24 h. IL-6 (Biolegend, San Diego, CA, USA), TNF-α (R&D system, Minneapolis, MN, USA), IL-1β (R&D System, Minneapolis, MN, USA), and PGE_2_ (R&D system, Minneapolis, MN, USA) in the supernatants were measured using ELISA kits following the manufacturer’s experimental protocols. The assay was performed at room temperature and the optical absorbance was measured at 450 nm using an ELISA microplate reader (ELx808) within 30 min.

### Griess assay

The NO in the culture supernatant was measured using Griess Reagent (1 % sulfanil-amide in 2.5 % H_3_PO_4_, 0.1 % N-(1-naphthyl)-ethylendiamine dihydrochloride). The cell culture supernatant was blended with Griess Reagent for 30 min, and the absorbance was read at 570 nm using an ELISA microplate reader (ELx808).

### DNA transfection and NF-kB reporter assays

Transient transfection of a reporter plasmid, pNF-kB-SEAP (Clontech Laboratories, Inc., Palo Alto, CA, USA) was performed for 293T cells seeded at 1 × 10^4^/well in a 96-well plate using Lipofectamine 3000 (Invitrogen, Carlsbad, MA, USA). One day after transfection, the cell medium was replaced with fresh DMEM and treated with ChondroT or GHJTY (0.3 and 0.1 mg/mL) for 4 h. The cells were treated overnight with PMA (Sigma Co., St. Louis, MO, USA) at a concentration of 1 ng/mL. The supernatants were incubated with QUANTI-Blue (Invitrogen, Carlsbad, MA, USA) for 2–4 h, and the absorbance was read at 630 nm with an ELISA microplate reader (ELx808).

### DPPH radical scavenging activity

Radical scavenging activity was measured using 2, 2-diphenyl-1-picrylhydrazyl (DPPH) (Sigma Co., St. Louis, MO, USA) and butylated hydroxyanisole (BHA) (Sigma Co., St. Louis, MO, USA) and vitamin C (VtC) (Sigma Co., St. Louis, MO, USA) were used as positive anti-oxidant drugs. ChondroT and other drugs dissolved in methanol were mixed with DPPH (0.15 mM) in a 96-well plate at room temperature for 30 min. The decrease in absorbance was measured at 470 nm using an ELISA microplate reader (ELx808).

### Statistical analysis

All studies were repeated at least three times. Statistical differences were evaluated using one way ANOVA. *P* value <0.05 was considered significant.

## Results

### The quality assessment of seven marker components in ChondroT

The HPLC-PDA method was developed for simultaneous determination of the quality assessment of seven marker components in ChondroT, which was composed of five medicinal herbs, *Ostericum koreanum* Maximowicz (Osterici Radix), *Lonicera japonica* Thunberg (Lonicerae Folium), *Angelica gigas* Nakai (Angelicae Gigantis Radix), *Clematis mandshurica* Ruprecht (Clematidis Radix), and *Phellodendron amurense* Ruprecht (Phellodendri Cortex). In this study, the seven compounds, oxypeucedanin hydrate from Osterici Radix, chlorogenic acid from Lonicerae Folium, nodakenin, decursin, and decursinol angelate from Angelicae Gigantis Radix, isoferulic acid from Clematidis Radix, and berberine Cl from Phellodendri Cortex were selected as marker compounds for quality control of ChondroT. The calibration curves of the seven marker components showed good linearity with a correlation coefficient (*r*^2^) ≥ 0.9996 in the different concentration ranges, and the other parameters were shown in Table 3. Using optimized chromatography conditions, the seven marker compounds were separated within 30 min. The typical HPLC chromatogram of ChondroT is shown in Fig. 1b and 1c. The retention times of the seven components, chlorogenic acid, berberine Cl, nodakenin, isoferulic acid, oxypeucedanin hydrate, decursin, and decursinol angelate were 8.94, 10.80, 12.00, 12.86, 15.95, 26.02, and 26.24 min, respectively. The amounts of the seven compounds were 0.81–5.46 mg/g, and the results are summarized in Table 4.

**Table 3:** Regression equations, linearity, LOD, and LOQ of the 7 marker compounds

Compound	Linear range (μg/mL)	Regression equation^a^	Coefficient of determination	LOD^b^ (μg/mL)	LOQ^c^ (μg/mL)
Chlorogenic acid	1.56–100.00	*y* = 30,420.94*x* – 42,881.66	0.9996	0.03	0.10
Berberine Cl	0.78–50.00	*y* = 81,886.13*x* + 16,338.31	0.9999	0.01	0.02
Nodakenin	0.78–50.00	*y* = 43,427.95*x* + 6,116.21	0.9999	0.02	0.06
Isoferulic acid	0.78–50.00	*y* = 44,822.70*x* – 7,991.61	1.0000	0.02	0.07
Oxypeucedanin hydrate	1.56–100.00	*y* = 26,221.17*x* + 5,076.06	1.0000	0.04	0.12
Decursin	0.78–50.00	*y* = 37,915.21*x* + 52,952.92	1.0000	0.04	0.12
Decursinol angelate	0.78–50.00	*y* = 28,967.64*x* + 29,015.53	1.0000	0.05	0.16

^a^ *y*: peak area (mAU) of compounds; *x*: concentration (μg/mL) of compounds

^b^LOD = 3 × 3.3 × standard deviation of blank / slope of regression

^c^LOQ = 10 × standard deviation of blank / slope of regression

**Table 4:** Amounts of the seven marker components in the ChondroT by HPLC (*n* = 3)

Compound	Mean (mg/g)	SD × 10^−1^	RSD (%)	Source
Chlorogenic acid	5.46	0.33	0.61	*L. japonica*
Berberine Cl	1.87	0.29	1.56	*P. amurense*
Nodakenin	1.70	0.18	1.06	*A. gigas*
Isoferulic acid	1.64	0.58	3.53	*C. chinensis*
Oxypeucedanin hydrate	2.03	0.33	1.65	*O. koreanum*
Decursin	1.09	0.02	0.16	*A. gigas*
Decursinol angelate	0.81	0.01	0.15	*A. gigas*

### Effects of ChondroT on the proliferation of SW1353 cells

**Fig. 2:**
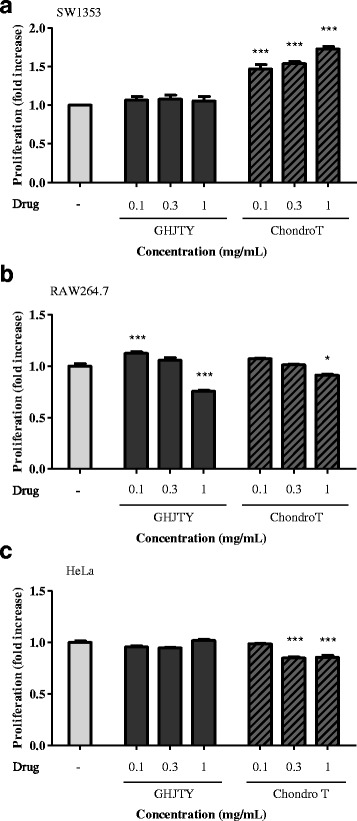
Effects of ChondroT on the proliferation of SW1353 chondrocyte cells. **a** SW1353 cells were exposed to GHJTY or ChondroT for 2 days, and the cell proliferation was assayed by MTS. ChondroT increased the proliferation of SW1353 cells at concentrations of 0.1 ~ 1.0 mg/mL. HeLa cells (**b**) or RAW264.7 cells (**c**) were treated with GHJTY or ChondroT for 2 days, and the cell proliferation was assayed by MTS. ChondroT did not show any cytotoxic effect on HeLa and RAW264.7 cells. ^*^ *P* < 0.05 and ^***^ *P* < 0.001 compared to the untreated group

The most predominant pathological change during osteoarthritis is cartilage degradation. To evaluate the effect on cartilage protection, SW1353 cells were treated with ChondroT or GHJTY for 2 days, and the cell proliferation was evaluated by MTS. ChondroT significantly increased the proliferation of SW1353 cells at concentrations of 0.1 ~ 1.0 mg/mL (Fig. 2a). In contrast, ChondroT and GHJTY did not show any cytotoxic effect on other origin cells, RAW264.7 and HeLa cells, at concentrations of 1, 0.3, and 0.1 mg/mL (Fig. 2b and 2c). This result suggests that ChondroT has specific proliferation activity on human-born fibroblast SW1353 cells.

### Effects of ChondroT on MMP1 expression in IL-1β- or PMA-induced SW1353 cells

**Fig. 3:**
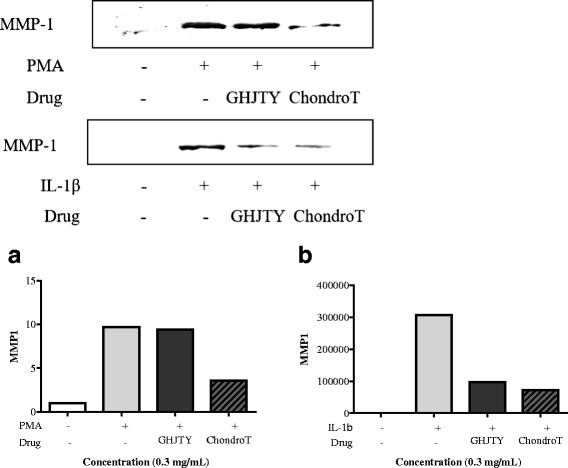
Effects of ChondroT on MMP1 expression in IL-1β- or PMA-activated SW1353 cells. SW 1353 cells were pretreated with or without ChondroT or GHJTY at a concentration of 0.3 mg/mL for 1 h and were then stimulated with PMA (**a**) or IL-1β (**b**). After 24 h, the MMP1 level was detected in cell culture supernatants using Western blot analysis. ChondroT reduced MMP1 expression in IL-1β- or PMA-activated SW1353 cells

MMPs play a critical role in cartilage destruction in arthritic joints, and MMP-1 is known to decompose a major component of chondrocytes. The effect of ChondroT was evaluated on MMP1 expression in SW1353 cells induced by IL-1β or PMA using Western blotting. MMP-1 expression was significantly increased by IL-1β or PMA in SW1353 cells, which was decreased by pretreatment with ChondroT (Fig. 3). ChondroT showed inhibitory effect on MMP1 expression in SW1353 cells greater than GHJTY (Fig. 3).

### Effects of ChondroT on the expression of COX-2 and iNOS in LPS-activated RAW264.7 cells

**Fig. 4:**
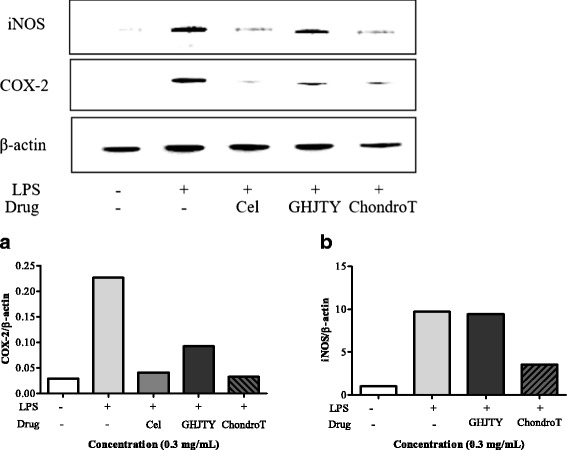
Effects of ChondroT on the induction of COX-2 and iNOS in LPS-activated RAW264.7 cells. RAW264.7 cells were pretreated with ChondroT for 2 h, and then LPS was added to the cells for 18 h. Beta actin was used as the control protein. The protein levels of COX-2 (**a**) and iNOS (**b**) were detected by Western blot analysis. Celecoxib (Cel) and ChondroT reduced the expression of COX-2 and iNOS in LPS-activated RAW264.7 cells

Proinflammatory enzymes, such as COX-2 and iNOS, cause pain and inflammation during arthritis. RAW264.7 cells were pretreated with GHJTY or ChondroT and LPS was added to the cells for 24 h. ChondroT significantly reduced the expression of COX-2 and iNOS in LPS-activated RAW264.7 cells similar to celecoxib (Cel), a COX-2 inhibitor (Fig. 4). The inhibitory effect of ChondroT on COX-2 and iNOS expression was greater than that exhibited by GHJTY (Fig. 4).

### Effects of ChondroT on the production of inflammatory mediators in LPS-activated RAW264.7 cells

**Fig. 5:**
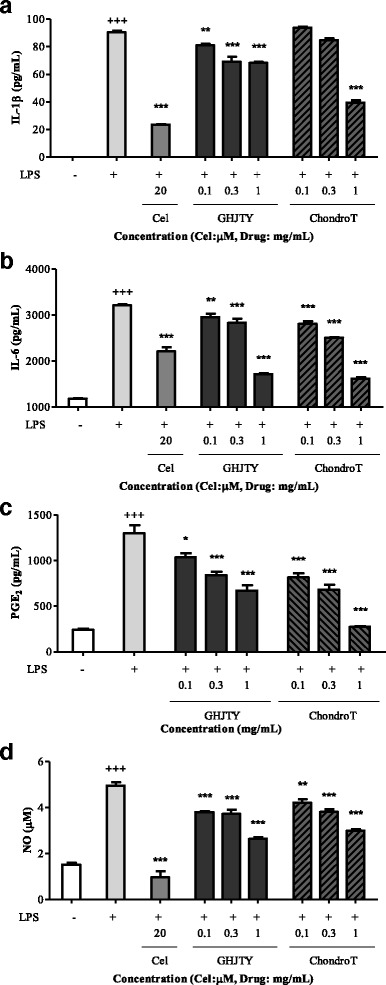
Effects of ChondroT on the production of pro-inflammatory cytokines in LPS-activated RAW264.7 cells. RAW264.7 cells were pretreated with ChondroT for 2 h and then incubated with LPS for 24 h. Inflammatory mediators were measured in the cell supernatants by ELISA. ChondroT decreased the LPS-induced production of IL-1β (**a**), IL-6 (**b**), and PGE_2_ (**c**). NO in the supernatant was detected by Griess reagent (**d**). LPS-induced NO production in RAW264.7 cells was decreased by ChondroT treatment. ^+^ *P* < 0.01 and ^+++^ *P* < 0.001 compared with the untreated group. ^*^ *P* < 0.05, ^**^ *P* < 0.01, and ^***^ *P* < 0.001 compared with the LPS-treated group

RAW264.7 cells were pretreated with ChondroT or GHJTY for 2 h prior to addition of LPS (500 ng/mL). IL-1β, TNF-α, IL-6, and PGE_2_ cytokines were assayed using ELISA kits. ChondroT significantly decreased the production of IL-1β, IL-6, and PGE_2_ in RAW264.7 cells activated with LPS (Fig. 5a, b, and c). NO production in LPS-activated RAW264.7 cells was also decreased by the pretreatment of ChondroT (Fig. 5d). The inhibitory effect of ChondroT on inflammatory mediator production was greater than that exhibited by GHJTY.

### Effects of ChondroT on PMA-induced NF-kB activation in 293T cells

**Fig. 6:**
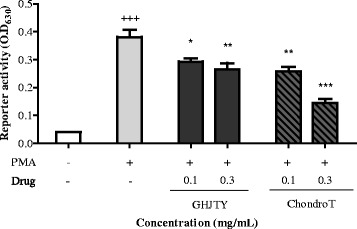
Effects of ChondroT on PMA-induced NF-kB activation in 293T cells. The 293T cells were transfected with a reporter plasmid, pNF-kB-SEAP. PMA-induced NF- kB transcription, which was inhibited by ChondroT at a concentrations of 0.3 and 0.1 mg/mL. ^*^ *P* < 0.05 and ^**^ *P* < 0.01 compared with the PMA-treated group

NF-kB is a transcription factor related to inflammation and arthritis. The effect of ChondroT was tested on NF-kB activation in 293T cells. PMA increased NF-kB transcription, which was decreased by ChondroT pretreatment (Fig. 6). The inhibitory effect of ChondroT on PMA-induced NF-kB activation was greater than that exhibited by GHJTY.

### Anti-oxidant activity of ChondroT

**Fig. 7:**
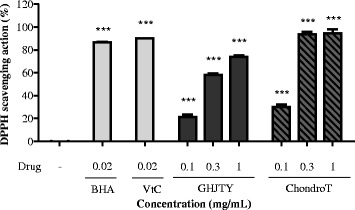
Anti-oxidant activity of ChondroT. ChondroT and other drugs dissolved in methanol were mixed with DPPH (0.15 mM in methanol) in 96-well plate for 30 min and then the absorbance was measured at 470 nm using an ELISA microplate reader. BHA and vitamin C were used as positive anti-oxidant drugs. DPPH free radicals were decreased by approximately 95 % at 0.3 mg/mL ChondroT, and the inhibitory effect was greater than that exhibited by GHJTY. ^***^ *P* < 0.001 compared with the control group

To study the anti-oxidant activity of the ChondroT, the DPPH scavenging potential was determined. Vitamin C and BHA were used as the positive controls. DPPH free radicals were decreased by approximately 95 % at 0.3 mg/mL ChondroT and the inhibitory effect was greater than that exhibited by GHJTY (Fig. 7).

## Discussion

In this study, we evaluated the multifunctional effects of ChondroT, a new complex herbal medication, for arthritis pharmacologic treatment. ChondroT is a water extract of 5 herbs, Osterici Radix*,* Lonicerae Folium, Angelicae Gigantis Radix, Clematidis Radix, and Phellodendri Cortex. We previously reported that Gangwhaljetongyeum (GHJTY), a traditional Korean herbal medicine comprised with 18 herbs, attenuates synoviocyte proliferation and reduces the production of proinflammatory mediators in macrophages [14]. GHJTY as an anti-arthritic drug may be limited because it is composed of 18 plants. To reduce the numbers of plants and to increase the potency as multifunctional anti-arthritic drugs, we conducted bioinformatics analysis [15]. In addition, oriental clinical doctors also suggested 4 complex herb medication candidates according to their clinical experiences. We compared the inhibitory effects of GHJTY and 4 complex herb medications on COX-2 and iNOS expression. Among them, an herb material ChondroT showed the most potent activity in chondrocyte protective effects and anti-inflammatory effects. ChondroT enhanced the proliferation chondrocytes (Fig. 2a) and also significantly inhibited IL-1β- or PMA-induced MMP-1 expression in the chondrocytes (Fig. 3). In addition, ChondroT decreased the expression of inflammatory enzymes COX-2 and iNOS (Fig. 4) and reduced the production of inflammatory mediators, such as IL-1β, IL-6, PGE_2_, and NO, thereby playing important roles in arthritis (Fig. 5). ChondroT also decreased the PMA-induced activation of NF-kB, a transcription factor related to inflammation and arthritis (Fig. 6). These results show that ChondroT exerted a chondroprotective effect and demonstrated a multi-target mechanism on inflammation and arthritis.

Oxidative stress was reported to induce apoptic cell death of chondrocytes and excessive production of various inflammatory cytokines in osteoarthritis, which further promote the expression of MMPs [16, 17]. Polysaccharide from Angelica sinensis was reported to protect chondrocytes from H_2_O_2_-induced apoptosis through its antioxidant effects in vitro [18]. The anti-oxidant effects of ChondroT (Fig. 7) can help patients suffering arthritis. These results suggest that ChondroT composed of 5 herbs has therapeutic potential for the treatment of arthritis.

Arthritis has become a significant clinical problem worldwide with an increase in the aging populations. NSAIDs and selective COX-2 inhibitors are used for pharmacologic treatment of arthritis. Recent study showed that celecoxib, a COX-2 inhibitor decreased NO production in chondrocytes from osteoarthritic rat joints and reduced inflammation by blocking NF-kB activation in a murine model [19–22]. However, some patients treated with these drugs complained of side effects such as gastrointestinal, cardiovascular, and other complications. Our results show that the suppressive effect of ChondroT on COX-2 and iNOS expression was similar that exhibited by celecoxib, a COX-2 inhibitor used for the treatment of arthritis (Fig. 4). In addition, ChondroT did not show any cytotoxicity to various origin cells (Fig. 2a, b, and c). Traditional herb medications have been concerned for the treatment of arthritis. Recently, SKI306X (Joins), an oriental herbal mixture were developed for osteoarthritis patients [1, 23–25].

ChondroT is a water extract of 5 herbs, Osterici Radix*,* Lonicerae Folium, Angelicae Gigantis Radix, Clematidis Radix, and Phellodendri Cortex. Effect of Phellodendri Cortex was reported in protecting human osteoarthritic and cartilage and chondrocytes [26]. Anti-inflammatory effects of ChondroT can be attributed to the action of all 5 herbs [27–31]. We evaluated the remedial value of ChondroT compared with GHJTY. The suppressive effect of ChondroT was greater than that exhibited by GHJTY, and it showed multifunctional therapeutic effects on inflammation and arthritis. For standard validation of ChondroT, a convenient and accurate HPLC–PDA detection method was used for simultaneous determination of seven reference components. In conclusion, ChondroT treatment increased chondrocyte proliferation, in part through a reduction in oxidative damage. In addition, ChondroT attenuated the severity of cartilage degradation factor. With its anti-inflammatory and anti-oxidative properties, ChondroT may constitute a promising therapeutic option for the management of arthritis.

## Conclusion

ChondroT exerted a chondroprotective effect and demonstrated a multi-target mechanism involving effects on inflammation and arthritis. In addition, the suppressive effect was greater than that exhibited by GHJTY, suggesting that ChondroT, a new complex herbal medication, has therapeutic potential for the treatment of arthritis.

## Abbreviations

BHA, butylated hydroxyanisole; Cel, celecoxib; COX-2, cyclooxygenase-2; DMEM, Dulbecco Modified Eagle’s medium; DPPH, 2,2-diphenyl-1-picrylhydrazyl; ELISA, enzyme-linked immunosorbent assay; FBS, fetal bovine serum; GHJTY, Ganghwaljetongyeum; HPLC–PDA, high-performance liquid chromatography–photodiode array; IL-1β, interleukin 1 beta; IL-6, interleukin 6; iNOS, inducible nitric oxide synthase; LPS, lipopolysaccharides; MMP1, matrix metalloproteinase 1; MTS, 3-(4, 5-dimethylthiazol-2-yl)-5-(3-carboxymethoxyphenyl)-2-(4-sulfophenyl)-2H-tetrazolium; NF-kB, *nuclear factor* kappa B; NO,nitric oxide; NSAIDs, nonsteroidal anti-inflammatory drugs; PGE_2_, prostaglandin E_2_; PMA, phorbol 12-myristate 13-acetate; PVDF, polyvinylidene fluoride membranes; TBST, tris-buffered saline and Tween 20; TNF-α, tumor necrosis factor-alpha; VtC, vitamin C
